# The COVID-19 Pandemic: A Longitudinal Study on the Emotional-Behavioral Sequelae for Children and Adolescents with Neuropsychiatric Disorders and Their Families

**DOI:** 10.3390/ijerph18189880

**Published:** 2021-09-19

**Authors:** Alessia Raffagnato, Sara Iannattone, Benedetta Tascini, Martina Venchiarutti, Alessia Broggio, Silvia Zanato, Annalisa Traverso, Cataldo Mascoli, Alexa Manganiello, Marina Miscioscia, Michela Gatta

**Affiliations:** 1 Department of Women’s and Children’s Health, Padua University Hospital, 35128 Padua, Italy; alessiaraffagnato@aopd.veneto.it (A.R.); benedetta.tascini@aopd.veneto.it (B.T.); martinavenchiarutti1489@gmail.com (M.V.); alessia.broggio@studenti.unipd.it (A.B.); silvia.zanato_01@aopd.veneto.it (S.Z.); annalisa.traverso@aopd.veneto.it (A.T.); cataldo.mascoli@studenti.unipd.it (C.M.); alexa.manganiello@studenti.unipd.it (A.M.); marina.miscioscia@unipd.it (M.M.); 2 Department of Developmental Psychology and Socialization, University of Padua, 35131 Padua, Italy

**Keywords:** mental health disease, psychiatric outpatients, children and adolescents, stress, resilience, parenting, COVID-19

## Abstract

This study aimed to investigate the immediate and short-term impact of the pandemic on the psychological well-being of Italian children and adolescents with psychiatric disorders and their families. Overall, 56 patients aged 6–18 (M = 13.4 years, SD = 2.77) and their parents were evaluated during the COVID-19 lockdown (T0) and after 4 months (T1). An ad hoc data sheet, Youth Self-Report 11–18 (YSR), Child Behavior Checklist 6–18 (CBCL), and Depression Anxiety Stress Scale-21 (DASS-21) were administered. Patients, mainly suffering from internalizing disorders, overall demonstrated a good adaptation to the pandemic context. Moreover, patients with behavioral disorders showed a greater psychological discomfort at both T0 and T1 compared to patients with internalizing disorders. Over time, patients presented an improvement on the emotional side, as proven by a significant decrease in internalizing and post-traumatic stress problems. Finally, no significant differences were found in the emotional-behavioral profile of patients according to the means of conducting neuropsychiatric interventions during the lockdown (i.e., in person/remotely/interrupted), thus allowing us to exclude important negative effects caused by the transition to remote therapy. Concerning parents, an inverse relationship emerged between the DASS-21 scores and the level of resilience, which therefore represents a protective factor against psychological maladjustment. Over time, an improvement in the psychological well-being of parents was observed, as shown by a significant decrease in mothers’ anxiety and fathers’ stress.

## 1. Introduction

After an outbreak of pneumonia of unknown etiology in the city of Wuhan in China, the World Health Organization (WHO) declared the epidemic of the novel coronavirus SARS CoV-2 (COVID-19) a public health emergency of international concern on 30 January 2020. The following day, in Italy, the Council of Ministers ordered several measures to deal with the emergency nationwide. Then, on 24 February 2020, more severe measures were applied, such as school closures and quarantine for all Italian citizens. On 11 March 2020, the coronavirus disease (COVID-19) was declared a global pandemic by the WHO [1]. To date, there have been almost 128 million positive diagnoses since its onset and 2.8 million dead [2]. As a result, specific plans were drawn up internationally to contain the virus and reduce the risk of infection. Among the measures taken, mass quarantine and general closure of commercial activities represented the most frequently applied, with about 4 billion people placed in lockdown in April 2020 [3]. Northern Italy was hit hard by the virus, especially in the early stages of its spread.

Though effective in containing the virus, the social isolation imposed by quarantine was demonstrated to have negative effects on the psychological well-being of the general population. In fact, research during previous outbreaks (e.g., SARS, MERS, Ebola) reported evidence of increased levels of anxiety [4], depression [5], stress [5], irritability [6], insomnia [6], and symptoms associated with post-traumatic stress [7], and such data were substantially confirmed during the current pandemic emergency [8]. In particular, excessive levels of stress can lead, in the long term, to the onset of psychiatric comorbidities [9] (anxiety disorders, depression, substance abuse)—especially in females [10]—and contribute to the acceleration of the pathological course or the exacerbation of chronic clinical conditions [11,12]. For instance, a case-control study [13] conducted in February 2020 in China compared a group of 76 psychiatric patients with 109 healthy subjects; results highlighted significantly higher levels of anxiety, depression, stress, suicidal thoughts, and insomnia in the first group. In addition, more than 40% of patients with mental disorders showed symptoms related to PTSD.

Since the outbreak of the pandemic, these results have been further reinforced by numerous studies conducted on large population samples [14,15,16], which have highlighted certain categories particularly at risk for the development of psycho-behavioral sequelae. In particular, children and adolescents are among the most exposed to such threats since many indispensable developmental needs have been severely limited by the pandemic control measures [16]. The lockdown period was characterized by a dangerous coexistence of risk factors such as: massive stress, reorganization of daily and family routine, fear of death and concern for loved ones, economic crisis, loss of support systems, limited access to hospital services, and reduced interaction with peers and teachers [17,18]. Several studies conducted on the pediatric population showed that emotional symptoms and self-regulation difficulties increased as a result of a breakdown in daily routines [19,20,21]. Moreover, a previous study [22] pointed out that the confinement measures and changes in daily routine negatively affected parents’ psychological dimensions, thus exposing children to a significant risk for their relational well-being.

In particular, children who already suffered from psychological problems aroused apprehension [22,23,24]. When a condition of pre-existing mental vulnerability during the developmental age—which represents a peculiar fragility itself—is impacted by strong stress, changes in daily habits, and loneliness, it may evolve towards more serious clinical conditions [25,26,27,28]. Therefore, children with psychiatric disorders and their caregivers confined to their homes in a situation such as the COVID-19 pandemic may experience an exacerbation of their condition. Following the lockdown measure, the Young Minds Institute in the UK conducted a study involving 2111 participants under the age of 25 with a previous history of mental illness; results showed that 83% perceived a deterioration in their clinical condition, and 26% of them were unable to access treatment services [29]. Moreover, a review of the literature on psychiatric disorders in adolescents during the COVID-19 pandemic confirmed an increase in the symptomatology in children with psychopathology, especially in those suffering from depression, ADHD, autism spectrum disorder, and eating disorder [25]. In addition, issues such as self-harm are related per se to social isolation and withdrawal [30], which can in some ways favor a progression from ideation to realization of suicidal self-harming [31]; at the same time, it is known how crucial the dynamics of family interaction within the NSSI phenomenon are [32]. On the other hand, it was shown that the resilience of the entire family has a mutual influence on mental well-being and the ability to successfully cope with changes, both in parents and children, resulting in stress and depression of children related to those of parents [22,33].

Furthermore, it should be considered that the COVID-19 pandemic caused temporary closures of or limitations to access to health services [23]. As a result, many patients interrupted therapies and were unable to receive the support they needed, with serious consequences on their psychological well-being [23]. Mental health care services also suspended most of their in-person activities, except in emergency situations, with a switch to interventions mediated by computer [34]. In particular, in the field of developmental psychiatry, the current coronavirus emergency has imposed the need to reorganize clinical and care activities supported by Italian policies; therefore, a massive recourse to telepsychiatry occurred in an effort to ensure continuity in the provision of care for patients and family, while also minimizing the risk of spreading the virus. The Italian Society of Neuropsychiatry of Childhood and Adolescence has produced practical guidelines [35] stating that, for children and adolescents with neuropsychiatric disorders and their families, the coronavirus emergency and consequent restrictions are a cause of severe overload, thus carrying a high risk for negative sequelae over time, including post-traumatic stress disorder. Our activities should therefore be reorganized with a focus on these issues. In practical terms, in our hospital, face-to-face meetings are generally replaced with more frequent and shorter audio or video conference calls, in order to monitor any changes, and to ensure that we adequately adjust our interventions, reprogram our service’s general activities, and coordinate our operators. Some works have highlighted that online assessment with young people has several advantages; in fact, it enables one to reach in a limited time a greater number of subjects at risk for developing psychopathology, while also remaining adherent to the indications given to reduce the spread of the contagion [33].

Children and adolescents with neuropsychiatric problems were also negatively affected by the suspension of in-person teaching, which represented a further obstacle to receiving the support and acquiring the personal resources they usually gain through their school routine [24]. A longitudinal study carried out during the spring 2020 lockdown showed a reduced emotional investment in school with a decrease in motivation to carry out tasks [36]. Children with difficulties in executive functions [37], predisposition to boredom, and low levels of self-control may have more difficulties with Distant Learning (DL), although remote school lessons can favor a personalization of teaching [38] and greater flexibility in the organization of tasks [37]. During DL, parents also experienced higher levels of stress, which may depend on family functioning, parental self-efficacy perception [39], and the support provided by the school, particularly in the cases of school-supported children [37].

For some groups of children with psychiatric diseases, such as those suffering from social anxiety disorders or depressive syndromes, mass quarantine has exacerbated the previous aspects of withdrawal, apathy, and social avoidance [24]. According to experts [25], there are some groups of patients who may suffer more intensely from the effects of prolonged isolation, such as children with neurodevelopmental disorders [40,41] or with eating disorders [42].

The present work is therefore part of a broader context of great concerns about the mental condition of children, following the dramatic events that occurred in the course of 2020. However, there is still a paucity of literature on this topic specifically targeted on children and adolescents with psychiatric disorders; in fact, several studies investigated the costs, in terms of mental health, of mass quarantine only in the general population.

### Study Aims and Hypotheses

Based on the above premises, this study aimed to explore the effects of the COVID-19 pandemic and the related containment measures on the mental health of young Italian patients with psychiatric diagnoses and their parents.

Therefore, the purpose of this work was twofold:

(a) To investigate the psychological well-being of children and adolescents with psychiatric disorders during the COVID-19 lockdown (T0) and after 4 months (T1). Specific attention was paid to variations in routine, habits, and biological functions, and stress-related symptoms. Moreover, given that an extensive shift to telepsychiatry occurred during the lockdown—with several experts expressing concerns about its potentially negative effects on mental health (e.g., [23])—we aimed to find out if the means of conducting neuropsychiatric interventions (i.e., continued in person, continued remotely, suspended) significantly affected the psychological adjustment of patients. Subsequently, since in the clinical practice a greater psychological discomfort was observed in children/adolescents suffering from disorders with predominantly behavioral expression (i.e., neurodevelopmental and/or conduct disorders), these patients were compared to those with internalizing symptomatology, to verify the hypothesis of worse outcomes for the former [40]. Finally, changes over time (T0–T1) in the psycho-behavioral profile of patients were monitored; considering that the retest was conducted during the least critical phase of the pandemic in Italy, we expected to detect an improvement in the symptomatology.
(b) To evaluate the psychological well-being of parents of children/adolescents with neuropsychiatric disorders during the COVID-19 lockdown (T0) and after 4 months (T1), also checking its change over time. Particular attention was paid to stress-related symptoms and elements of resilience. Specifically, according to the literature, an inverse relationship between psychological maladjustment and resilience was hypothesized [22,43]. Then, we expected to observe a decrease in anxiety, depression, and stress symptoms of parents over time, following the easing of the COVID-19 containment measures.

## 2. Materials and Methods

### 2.1. Participants and Recruitment Criteria

Patients were selected according to the following inclusion criteria:

- Individuals with a psychiatric disorder already monitored at the Neuropsychiatric Unit of the Padua University Hospital (Italy) and receiving pharmacological, psychiatric, and/or psychological treatments, or undergoing psychodiagnostic assessment in March 2020.
- Age between 6 and 18 years.

The exclusion criteria were:

- Presence of factors that compromise the correct interpretation and compilation of the administered tests, such as severe intellectual disability and important language barriers.
- Psychiatric acute situations and severe decompensation of the disease at the time of recruitment.

On the basis of the above criteria, among 73 potentially recruitable patients, 17 subjects were excluded from the study, including 9 boys and 8 girls.

At T0, the sample consisted of 56 subjects (and their families), of which 38 (67.9%) were girls and 18 (32.1%) boys, with an average age of 13.4 years (SD = 2.77). More specifically, 9 (16.1%) patients were between 6 and 10 years old, 24 (42.9%) were between 11 and 14 years old, and 23 (41.1%) were between 15 and 18 years old.

In the second phase of the study (T1), 17 families decided to drop out. The occurrence of dropping out was partly due to the pandemic context and the restrictive measures that made it more difficult to travel and/or to maintain active, even telematic, psychiatric assistance; in two cases, dropping out was influenced by the transfer of the children to a psychiatric residential structure. Therefore, at T1 the sample consisted of 39 patients and their parents. Among children and adolescents with psychiatric disorders, 25 were girls (64.1%) and 14 were boys (35.9%), with an average age of 13.3 years (SD = 2.84). To be specific, 6 (15.4%) patients were between 6 and 10 years old, 17 (43.6%) were between 11 and 14 years old, and 16 (41%) were between 15 and 18 years old.

### 2.2. Procedures

The study was conducted at an outpatients’ service of the Child and Adolescent Neuropsychiatry Unit in Veneto, Italy. The study was approved by the Ethics Committee of the Padua University Hospital (ref. Protocol 0031095, CESC n.25 of 22 April 2021), and written informed consent was obtained from all the subjects involved (i.e., children and their parents).

We selected a longitudinal research design since it is considered highly valid for examining changes over time in health-related behaviors and processes, especially in the field of developmental psychopathology (e.g., [44]). Therefore, given that we were interested in observing the effects over time of the COVID-19 pandemic on the mental state of young patients and their parents, we deemed such a research design the most suitable for the purpose of the study.

Firstly, a semi-structured interview and standardized questionnaires (see list below) were administered to patients and parents during April and the beginning of May 2020 (T0), corresponding to the period of implementation of the strictest social distancing measures in Italy, such as national quarantine.

The second administration (retest T1) was conducted during August and September 2020 (after 4 months); this second moment was distinguished by a gradual relaxation of the national measures against the spread of the virus.

At both T0 and T1, questionnaires were administered to patients remotely (i.e., via email or phone-call) or, when possible, in person, at the time of clinical interviews for their diagnostic assessment. Parents, instead, completed the survey remotely (i.e., via email).

### 2.3. Tools

*Information and data collection sheet*: This contains personal, familiar, and clinical details, and information about participants’ daily routines, stress indicators, and adaptation to the pandemic. 

Specifically, the following main variables were analyzed:

- Personal and social data of patients and parents: age of patients, sex, marital/parental status, single- or two-parent family, and socio-economic status (SES). This last one was determined by means of the SES index, which is based on the Hollingshead Four Factor Index of Social Status [45]. This is a tool that enables one to identify the socio-economic level of a person by attributing a score to his/her educational level and profession. Specifically, the educational level is measured on a 7-point Likert scale (1 = primary school, 7 = post-university), and the profession is classified on a 9-point Likert scale that places socially and economically poor jobs at the lowest levels, while those that are more qualified are at the highest levels. Then, a weighted total score is calculated, which provides five different socio-economic levels: low SES = score between 8 and 19, medium-low SES = score between 20 and 29, medium SES = score between 30 and 39, medium-high-SES = score between 40 and 54, high SES = score between 55 and 66.
- Neuropsychiatric problems: ICD-10 diagnoses [46] and any comorbidities.

To describe the sample of patients, nosographic definitions according to the ICD-10 code were grouped into three broader diagnostic categories:

(1) Emotional-affective disorders: affective syndromes with F30–F39 codes (bipolar disorder, depressive disorder) and phobic syndromes, syndromes linked to stress and somatoforms with F40–F48 codes.
(2) Disorders of behavior and personality: disorders of adult personality and behavior with F60–F69 codes, and behavioral and emotional disorders with onset usually occurring in childhood and adolescence with F90–F98 codes (such as the disorder of activity and attention, conduct disorder, and oppositional disorder).
(3) Neurodevelopmental disorders and psychosis: non-mood psychotic disorders with F20–F29 codes and disorders of psychological development (specific learning disorders and autism spectrum disorders) with F80–F89 codes.

Then, for the purpose of the study, a further subdivision into two main categories was used: the first category—called “internalizing”—includes emotional problems with little behavioral implications, as in most cases of anxious-depressive, bipolar, phobic, and somatoform syndromes (ICD-10 codes: F30–F48); the second category—called “behavioral”—includes disorders with predominantly behavioral expression, such as attention deficit hyperactivity disorder, oppositional disorder, conduct disorders and explosive-intermittent disorders, and autism spectrum disorders (ICD-10 codes: F20–F29, F80–F98). This last category also includes mixed forms, in which an emotional-affective problem also presents a behavioral expression (e.g., disorder of adult personality and behavior with F60–69 codes).

- Patients’ history of trauma (presence/absence, description of any prior trauma).
- Patients’ ongoing treatments before and during the pandemic (psychiatric, pharmacological, psychological, educational), and changes in the means of conducting these interventions during the lockdown (continued face-to-face or interrupted), specifying if maintained psychiatric interventions were conducted remotely or in person.
- Variations in patients’ biological functions (sleeping-waking rhythms, diet), body weight (measured in kg), general habits (e.g., physical activities), and exposure to screen-based media during the lockdown compared to the previous period.
- Stress-related symptoms in patients and parents, at both T0 and T1: physical symptoms (e.g., headache, stomachache, difficulty breathing, tachycardia, agitation, sleeping difficulties, loss of appetite), behavioral problems (e.g., teeth grinding, compulsive eating, frequent alcohol drinking, difficulty completing tasks), emotional symptoms (e.g., anger, irritability, anxiety, sadness, sense of powerlessness, demotivation) and cognitive symptoms (e.g., problems with making decisions, distraction/inattention, constant worry, lack of creative spirit). The total number of stress-related symptoms was taken into consideration, and four ranges were highlighted: 0 symptoms (range 0), between 1 and 5 symptoms (range 1), between 6 and 10 symptoms (range 2), and more than 10 symptoms (range 3).
- In the data collection sheet administered to parents, some ad hoc questions about resilience were added. To structure such questions, we considered the three components of psychological resilience described by Kosaba [47]: question (1)—commitment (I let myself be involved in activities, I am active, I have goals to achieve); question (2)—control (I do not feel at the mercy of events, I think I can dominate them, I am able to know when to act and when to stop); question (3)—challenge (I accept change and I think it’s an opportunity for growth, I see the bright side rather than the downside of it). Each question provides four possible ways to answer: never, sometimes, often, always. Based on the responses to each item, a level of resilience has been defined as “low” (never, sometimes) or “high” (often, always).

*The Achenbach questionnaires,* namely *Child Behavior Checklist 6–18 (CBCL)* and *Youth Self Report 11–18 (YSR)* [48], are widely used in clinical setting and research for rating juvenile behavior. Both CBCL and YSR have been validated in samples of young Italian patients and their parents, showing a good validity and reliability (α > 0.65) [49]. The CBCL was administered to all parents, and the YSR to adolescents over the age of 11. Both questionnaires consist of 112 items that define two profiles. One profile is related to competences (activities, social functioning, school performance), which informs about the child’s level of personal autonomy, social skills, and performances in sports, hobbies, and school. The other profile is related to behavioral and emotional problems, both of which can be assessed as “normal”, “borderline”, or “clinical” on eight specific syndrome scales relating to the various possible clinical pictures; the latter are in turn grouped into three problems scales: internalizing problems (anxiety, depression, withdrawal, somatization), externalizing problems (aggressive and rule-breaking behavior), and other problems (social problems, thought problems, attention problems), which are combined in the total problems subscale. For analyses carried out using the CBCL and YSR questionnaires, only the internalizing problems, externalizing problems, total problems, and post-traumatic stress (PTSP) subscales were considered.

*Depression Anxiety Stress Scale-21 (DASS-21)* [50,51] was given to caregivers. It is a self-report questionnaire which consists of 21 items measuring stress, anxiety, and depression. Each item is assigned Likert-scale responses from 0 to 3: 0 = never, 1 = sometimes, 2 = often, 3 = always. The original version of the tool has excellent internal consistency values, with Cronbach alpha values between 0.82 and 0.93. The Italian version [50] also shows excellent psychometric properties, with Cronbach alpha values for subscales ranging from 0.74 to 0.85. To score the questionnaires, the website of the Viterbo Association of Psychological Counseling and Research (ASCRIP) [52] was used.

### 2.4. Data Analysis

Data were analyzed with the Jamovi statistical program (version 1.6) [53].

Following the recommendations of the literature, non-parametric tests were used, given that they have proven to be excellent in controlling Type I error rates in cases of small sample size and non-normal distribution (e.g., [54,55]).

In the preliminary analyses, descriptive statistics and frequencies tables were calculated to delineate the socio-demographic and clinical characteristics of both patients and their parents. At T1, the clinical features of the sample (e.g., diagnostic categories, stress-related symptoms, etc.) were described in more detail.

Before performing the subsequent analyses, we checked whether the clinical subgroups of patients (at both T0 and T1), the means of conducting neuropsychiatric interventions during the lockdown, and the scores on the considered scales of YSR and CBCL (at both T0 and T1) significantly differed for age and gender. Since no significant differences emerged, and the literature strongly discourages the inclusion of several variables in the model when sample size is limited and group size is unequal (e.g., [56], we did not consider age and gender in the following analyses.

Subsequently, in order to find out if the means of conducting neuropsychiatric interventions during the lockdown (T0) (three levels: maintained in person, maintained remotely, or interrupted) affected the psycho-behavioral profile of patients, the Kruskal–Wallis test [57] was conducted. Specifically, the considered scales of YSR and CBCL were inserted as dependent variables, and the type of intervention as independent variable.

Then, the Mann–Whitney U test [58] was applied to check if there was a significant difference in the total problems and post-traumatic stress problems (PTSP) scales of YSR and CBCL based on the clinical subgroups of patients (internalizing vs. behavioral), considering both T0 and T1.

Concerning the relationship between parents’ resilience and their level of stress, anxiety, and depression, Spearman’s rho (ρ) correlations between the DASS-21 scales and questions about resilience were conducted, considering both T0 and T1. Spearman’s rho coefficients have been directly interpreted as a measure of effect size, according to Cohen’s [59] criteria: ρ = 0.10 (small), 0.30 (moderate), 0.50 (large).

Finally, to check if there was a significant change over time (T0 to T1) in the emotional-behavioral aspects of patients, the paired sample Wilcoxon rank test [60] was applied, entering as variables the scores on the YSR and CBCL scales. Along the same line, the paired sample Wilcoxon rank test was conducted, considering as variables the scores on the DASS-21 scales, in order to verify if there was a significant change over time in the mental well-being of parents.

Statistical significance was set at *p* < 0.05.

## 3. Results

### 3.1. Description of the Socio-Demographic and Clinical Characteristics of the Sample at T0

#### 3.1.1. Patients

With regard to the diagnostic classification of mental illness and behavior by nosographic criterion according to the ICD-10 [46], the sample of children and adolescents consisted of:

- 38 patients (67.9%) suffering from “emotional-affective disorders”, corresponding to clinical situations of mood disorders (ICD-10 F30–39, in particular depressive disorder, mixed affective disorder) and neurotic disorders, related to stress and somatoforms disorders (ICD-10 F40–48, including anxious disorders, mixed anxious-depressive, and obsessive-phobic disorders).
- 7 patients (12.5%) with “behavior and personality disorders”, with ICD-10 codes F60–69 and F90–98; personality disorders, attention deficit hyperactivity disorder (ADHD), conduct disorder (DC), and oppositional defiant disorder (PDO) are detected.
- 11 patients (19.6%) with neurodevelopment disorders and psychosis, with ICD-10 codes F80–89 and F20–29, such as autism spectrum disorders and psychotic syndromes.

Pertaining to the clinical division between presence and absence of behavioral expression, the sample consisted of 18 children (32% of the sample) with significant behavioral expression (behavioral) and 38 children (68% of the sample) with poor behavioral expression (internalizing). More specifically, the patients with predominantly behavioral problems were 8 boys (44.5%) and 10 girls (55.5%), with a mean age of 12.7 years (SD = 2.91); in this group, 15 patients (83.3%) were between 11 and 18 years old, and were thus the appropriate age to fill out the YSR. The patients in the internalizing category were 10 boys (26.3%) and 28 girls (73.7%), with a mean age of 13.7 years (SD = 2.68); in this group, 32 patients (84.2%) were between 11 and 18 years old, so they could complete the YSR.

As for the other clinical variables evaluated, 14 children (25%) with neuropsychiatric disorders experienced a previous traumatic event, defined as stress of considerable importance in the child’s life: this is represented in 42.9% of cases by a conflicting separation of parents, in 28.8% of cases by an inability to adapt to a new environmental context, in 21.4% of cases by loss or serious pathologies in the family context, and in 7.1% of cases (1 child only) by frequent episodes of prolonged hospitalization.

During home quarantine, 25% of families reported the exacerbation of some behavioral problems, such as more frequent and intense episodes of non-collaboration, indifference, physical/verbal aggression, poorly targeted/organized play, screaming/crying, social isolation, provocative attitudes towards others, attempts to escape, and self-cutting ideation.

Regarding daily routines and biological functions during the lockdown, 44.6% of patients did not report changes in general habits. Moreover, only 12.5% of patients reported changes in the use of electronic devices, 21.4% described an alteration in sleep-wake rhythm, 12% experienced a change in nutrition, and 10.7% had increased in weight. Therefore, the vast majority of patients did not experience changes in daily routines and biological functions.

With regard to stress-related symptoms, 53.6% of patients reported 0 symptoms of stress, 23.2% 1 to 5 symptoms of stress, 17.9% 6 to 10 stress symptoms, and 5.4% more than 10 stress symptoms. As can be noticed, the presence of stress-related symptomatology was not detected in more than half of patients.

Concerning drug treatment, 25 children had ongoing psychopharmacological treatment at the end of February (44% of the total), and this increased to 27 (48% of the total) as of March.

Pertaining to neuropsychiatric interventions, between the pre-lockdown phase (February 2020) and lockdown (from March 2020), among the 48 patients (86% of the total) with an active neuropsychiatric intervention at the end of February, 44 patients maintained the intervention (79%), 5 of which were face-to-face and 39 remotely.

Regarding psychological therapy, among the 31 children treated with psychotherapy in February 2020 (55% of the total), only 3 patients (9.7%) completely suspended this intervention during the lockdown.

Then, among the 10 children (17% of the total) who received educational-rehabilitation treatment at the end of February, no changes in this type of intervention were observed during the lockdown.

Finally, among patients and their parents, there was only one positive element to the virus: no hospitalization was experienced and only one case of quarantine took place in relatives. 

Table 1 displays the main YSR (*N* = 41) and CBCL (*N* = 55) scale scores at T0.

#### 3.1.2. Parents

The socio-demographic characteristics of parents (56 mothers and 56 fathers), referring to the lockdown period, are shown in Table 2.

With regard to stress-related symptomatology, 61% of mothers and 75% of fathers reported the absence of stress-related symptoms, 30% of mothers and 23% of fathers reported 1 to 5 symptoms, 5% of mothers and 2% of fathers reported 6 to 10 symptoms, and 4% of mothers reported a total number of stress symptoms greater than 10.

Concerning resilience, in question 1 related to commitment, 79.6% of mothers and 85.7% of fathers got a high score; in question 2 about control, 67.4% of mothers and 85.7% of fathers obtained a high score; and in question 3 about challenge, 67.4% of mothers and 77.6% of fathers reported a high score.

Table 3 shows mean scores obtained by mothers and fathers on the DASS-21 scales at T0.

### 3.2. Differences in the Psycho-Behavioral Profile of Patients According to Diagnostic Category and Type of Treatment at T0

The Kruskal–Wallis test, carried out to verify the effect of changes in neuropsychiatric interventions (kept in person, maintained remotely, interrupted) on patients’ psycho-behavioral profiles evaluated through YSR and CBCL, showed no significant results.

On the other hand, statistically significant results emerged from comparing the CBCL scores (total problems and PTSP scales) according to the clinical categories (i.e., internalizing vs. behavioral) by means of the Mann–Whitney test. Specifically, for maternal CBCLs, the clinical category was found to have a significant effect only on the total problem scale (U = 172, *p* = 0.004), while for paternal CBCLs, it had a significant effect on the total problem scale (U = 99.5, *p* < 0.001) and PTSP scale (U = 216.5, *p* = 0.037). To be more specific, higher scores were reported for patients with behavioral problems (CBCL mother total problems: M = 67.9, SE = 2.13; CBCL father total problems: M= 67.4, SE = 1.74; CBCL father PTSP: M = 69.7, SE = 2.44) compared to patients without such problems (CBCL mother total problems: M = 60, SE = 1.51; CBCL father total problems: M = 55.6, SE = 1.54; CBCL father PTSP: M = 63.6, SE = 1.57).

Finally, no statistically significant data emerged considering YSR.

### 3.3. Relationship between Parents’ Well-Being and Resilience at T0

To investigate the relationship between parents’ well-being and their resilience, Spearman’s ρ correlations were carried out (Table 4). As can be noticed, correlations were all negative. More specifically, for question 1 (“commitment”), significant and medium-high in magnitude correlations emerged with all the DASS-21 scales, except for the maternal anxiety scale (*p* = 0.12) and the paternal stress scale (*p* = 0.06). As for question 2 (“control”), low and non-significant correlations emerged only with the maternal DASS-21 stress scale (*p* = 0.14) and the paternal anxiety scale (*p* = 0.099), while the other correlations were all significant and moderate in magnitude. Finally, as regards question 3 (“challenge”), correlations were all significant; in particular, high correlations emerged with both maternal and paternal DASS-21 stress scales, while the other correlations were medium in magnitude. Overall, these results support an inverse relationship between resilience and psychological maladjustment.

### 3.4. Follow-Up 

#### 3.4.1. Description of the Clinical Features of the Sample at T1

Overall, 39 children and adolescents (and their parents) took part in the retest phase: 25 girls (64.1%) and 14 boys (35.9%), with an average age of 13.3 years (SD = 2.84). 

With regard to diagnostic categories (internalizing vs. behavioral), 25 (64.1%) patients were affected by internalizing disorders and 14 (35.9%) by behavioral disorders. Particularly, the patients suffering from behavioral problems were 7 (50%) boys and 7 (50%) girls, with a mean age of 12.4 years (SD = 3.16); in this group, 11 (78.6%) patients were between 11 and 18 years old, so they could complete the YSR. The internalizing problems category was composed of 18 (72%) girls and 7 (28%) boys, with a mean age of 13.8 years (SD = 2.70); in this group, 22 (88%) patients were of the right age to fill out the YSR (11–18 years).

As for their therapeutic regimen at T1, 26.47% of patients had a psychiatric treatment ongoing, 32.4% a drug therapy, 38.2% a psychological therapy, and 14.7% an educational-rehabilitative therapy.

Pertaining to the number of stress-related symptoms of children and adolescents, 44.4% reported 0 symptoms, 32.4% between 1 and 5, 14.7% between 6 and 10, and 8.8% more than 10 symptoms.

As regards the scores obtained on the YSR (*N* = 31) and CBCL (*N* = 39) scales at T1, these are presented in Table 5.

Considering parental environment at T1, among mothers, 47.1% reported 0 stress-related symptoms, 26.5% between 1 and 5 symptoms, 14.7% between 6 and 10 symptoms, and 11.8% more than 10 symptoms. Among fathers, 70.6% reported 0 symptoms, 20.6% between 1 and 5 symptoms, 2.9% between 6 and 10 symptoms, and 5.9% more than 10 symptoms.

The DASS-21 scale scores obtained by parents at T1 are shown in Table 6.

#### 3.4.2. Differences in the Psycho-Behavioral Profile of Patients According to Diagnostic Category at T1

Differences in the YSR and CBCL scores at T1 according to clinical categories (i.e., internalizing vs. behavioral) were evaluated by means of the Mann–Whitney test. A statistically significant difference between the two clinical categories emerged only in the paternal CBCL total problems scale (U = 106, *p* = 0.045). In particular, higher scores were reported for patients with behavioral disorders (M = 62.6, SE = 2.68) compared to patients with internalizing problems (M = 55, SE = 2.23). Therefore, as already pointed out at T0, a greater perception of psychopathology in association to behavioral expression remains at T1, too. Moreover, as found at T0, no differences on the basis of the two clinical categories emerged considering the YSR scales.

#### 3.4.3. Relationship between Parents’ Resilience and Well-Being at T1

Spearman’s ρ correlations showed an inverse relationship between the level of resilience reported in each question and the DASS-21 scores for both mothers and fathers (Table 7). Specifically, considering question 1 (“commitment”), the only significant correlation emerged with the paternal DASS-21 depression scale, while the other correlations were all not significant and medium-low in magnitude. As regards question 2 (“control”), significant and moderate correlations were found with the paternal DASS-21 anxiety and stress scales, while the other correlations were all not significant. Finally, concerning question 3 (“challenge”), low and non-significant correlations were found with the maternal DASS-21 depression and anxiety scales, while the other correlations were all significant and medium-high in magnitude.

Given that the statistical significance of a correlation is influenced by the size of the sample (e.g., [61,62]), the presence of more non-significant correlations at T1 than at T0 could be due to the reduction of the sample size at the retest phase (N_T0_ = 56 vs. N_T1_ = 39). For this reason, in interpreting results, we only considered the magnitude of a correlation, rather than its statistical significance, as suggested by the literature (e.g., [61,62]). Therefore, taken together, the results of the retest phase give further support to what previously emerged at T0, namely that a lower symptomatology of anxiety, depression, and stress is associated with a higher level of resilience.

#### 3.4.4. Change Over Time (T0–T1) in the Emotional-Behavioral State of Patients and Their Parents

Pertaining to change over time in the psycho-behavioral state of patients, the scores on the YSR and CBCL total problems, internalizing problems, externalizing problems, and PTSP scales in the second phase (T1) were compared, through the paired sample Wilcoxon rank test, with the scores obtained on the same scales as in the previous quarter (T0). The comparison showed a statistically significant reduction in scores on the maternal CBCL internalizing problems scale (W = 395, *p* = 0.042; M_T0_ = 66.9, SE = 1.67; M_T1_ = 64.2, SE = 1.67). There was also a reduction in T0 to T1 scores on the scale of post-traumatic stress problems (PTSP) in both mothers’ (W = 470, *p* = 0.003; M_T0_=68.9, SE = 1.77; M_T1_=64.9, SE = 1.54) and fathers’ CBCL (W = 570, *p* = 0.020; M_T0_ = 65.1, SE = 1.61; M_T1_ = 62.3, SE = 1.59). On the other hand, there were no statistically significant changes in YSR scores from T0 to T1.

With regard to changes over time in the psychological well-being of parents, the paired sample Wilcoxon rank test showed a statistically significant decrease in scores on the maternal DASS-21 anxiety scale (W = 166, *p* = 0.022; M_T0_ = 47.3, SE = 1.53; M_T1_ = 43.4, SE = 0.85) and the paternal DASS-21 stress scale (W = 195, *p* = 0.027; M_T0_ = 46.9, SE = 1.20; M_T1_ = 44.7, SE = 1.07).

## 4. Discussion

The present study aimed to investigate the immediate (T0) and short-term (T1) impact of the COVID-19 pandemic and quarantine on the psycho-behavioral profile of young psychiatric patients and their parents.

With regard to the psychological well-being of children and adolescents with mental health disorders, no major changes were observed during the lockdown compared to the previous period. In fact, although changes in general habits were detected in more than half of the sample (55.4%), a considerable proportion of patients did not report such changes, and the vast majority (80–90%) did not present variations in biological functions, body weight, or daily exposure to screen-based media. Moreover, most patients did not report any symptoms of stress at both T0 and T1. Taken together, these results suggest a good ability of patients to adapt to a new and difficult context. Particularly, it could be hypothesized that subjects with psychiatric disorders present a kind of resistance—or maybe “habit”—to disadvantaged mental and environmental conditions, which prepares them to face difficulties. In addition, as stated by Conti et al. [63]—who found a better reaction to quarantine in pediatric Italian patients with psychiatric disorders compared to patients with neurological or neurodevelopmental problems—the presence of a psychopathological diagnosis may have had a “protective effect” during the lockdown linked to the reduction of several sources of stress (e.g., schools or competition with peers).

Nevertheless, this finding and its interpretation may be somewhat limited by the lack of a control group composed of children and adolescents from the general population. Consequently, to provide a full picture of this important topic, further research comparing children with and without psychiatric disorders should be undertaken. Furthermore, in the interpretation of results, it is also necessary to consider the characteristics of the sample, mainly composed of patients with disorders in the emotional-affective sphere, thus characterized by aspects that the lockdown has supported and normalized, such as social and performance anxiety, tendency to withdrawal, and avoidance.

During the lockdown, a massive shift to telepsychiatry [34] has been foreseen, linked to the reorganization of care facilities due to the health emergency. Various experts expressed concerns about the often-abrupt change that remote therapy implies [23,64]; therefore, we investigated what therapeutic changes were introduced in our sample of patients, and whether there were consequences, in terms of psychological well-being, resulting from continuation (distinguishing between in person and remote) or suspension of neuropsychiatric treatments during the lockdown. Psychiatric therapy was completely suspended in only 8.3% of patients, and among those for whom it was maintained, 88% of the interventions were converted into telematics. This demonstrates an efficient logistical capacity of the structure to cope with the reorganizing requests dictated by the pandemic and the application of the SINPIA guidelines [35].

No statistically significant differences were found in the emotional-behavioral profile of our patients according to the type of ongoing neuropsychiatric treatment (i.e., in person/remote/interrupted). These data at least allow us to exclude important negative effects caused by the transition to remote therapy and appear to be in agreement with the arguments in favor of the use of telepsychiatry as a valid and effective methodology [17,65]. Nevertheless, this finding should be treated with caution, given that it derives from analyses performed on groups highly different in size.

Pertaining to the differences between patients belonging to the diagnostic category “internalizing” and those belonging to the diagnostic category “behavioral”, what has been observed, especially in clinical practice during the lockdown, is that children/adolescents with neurodevelopmental disorders and/or with externalizing disorders have been those most affected by the adopted restrictive measures. This clinical observation was then confirmed by higher scores in the CBCL total problems scale—which can be considered a general indicator of psycho-behavioral discomfort—in reports provided by both mothers and fathers of patients with behavioral problems. Moreover, a greater malaise for these patients was confirmed at T1, too. In fact, the CBCLs compiled by fathers report a greater presence of total problems for patients with behavioral problems compared to patients with internalizing problems. These results support the initial hypothesis that confinement within the home, and loss of rhythms, commitments, and planned therapeutic interventions may have represented significant obstacles for children/adolescents with externalizing problems [19]. In fact, in these patients, the tendency towards inflexibility and insistence on sameness, which are often distinctive features of the disorder itself [25,41], make the adaptation to a new situation difficult. In addition, the breakdown of routines, the impossibility of having a clear forecast of daily planning, the uncertainty related to the emergency situation, and the suspension of individual and group rehabilitation interventions may have contributed to accentuating some basic behavioral problems, thus also increasing the difficulties of parents in managing their children’s problem behaviors [17,24,25]. On the other hand, as regards patients with internalizing problems, it may be that forced isolation within the home made them feel less exposed to those social and performance triggers causing depression, anxiety, or stress, such as school, sport—especially if competitive—and situations where the child/adolescent is asked to relate appropriately to others. The lockdown also helped to temporarily break down the differences between the social isolation of these patients and that of their peers, somehow normalizing and standardizing it (at least in appearance).

Therefore, our results substantiate previous findings in the literature, according to which patients with externalizing disorders, such as attention deficit hyperactivity disorder and autism spectrum disorder, are the most vulnerable to the effects of prolonged isolation and quarantine [40,41]. For example, the Italian study by Bentenuto et al. [66] found an increase in externalizing problems during the lockdown in children with neurodevelopmental disorders. The authors also underlined that the increase in externalizing behaviors may be predicted by the reduction of therapeutic and rehabilitative interventions.

Nevertheless, contrasting results emerged from another Italian work, conducted with pre-school children [67]. This study showed a worsening of the scores on the CBCL during quarantine compared to the previous period, in terms of both externalizing problems, such as aggressive behavior and oppositional-provocative disorder, and anxious-depressive symptoms. However, this work involved children aged 2 to 6, both healthy and at familiar risk for neurodevelopmental disorders; therefore, the differences with our findings may be due to the different characteristics of the two samples.

Another noteworthy aspect concerns the differences between mothers and fathers in the scores on the CBCL. In fact, mothers reported higher CBCL scores on all the scales considered at both T0 and T1 compared to fathers. Discrepancies between the CBCL scores of mothers and fathers have been already described in the literature, with higher scores reported by the former (e.g., [68,69,70]). A possible explanation of this difference may be linked to parental stress, which is usually higher in mothers due to their primary caregiving role [68]. In particular, this role became more difficult during the lockdown since mothers had to conciliate time spent with their children, personal work, and family management [19]. Such conditions may have affected the perception of their children’ behaviors, which were therefore rated as being more severe.

With regard to change over time in the psycho-behavioral state of patients compared to the lockdown period, they presented in the following months an improvement on the emotional side, proven by a significant reduction in scores at the CBCL scales regarding post-traumatic stress problems and internalizing problems. This result confirms our initial hypothesis of an improvement in the symptomatology due to the easing of the COVID-19 containment measures during the least critical phase of the pandemic in Italy. Nonetheless, this finding should be carefully interpreted; in fact, all patients had ongoing treatment, which, therefore, may have contributed to improving their psychological well-being.

Another purpose of this work was to evaluate the psychological well-being of caregivers of children/adolescents with psychopathology during the different phases of the pandemic, also monitoring its change over time. The closure of schools, the strong limitations of social interactions, and the increasing of time spent together, also due to parents’ remote working, brought the family structure to a greater importance [71] for the mental well-being of children, at a time when the fallout in mental health may also afflict the adult population [72,73]. Parents are in fact essential to help children to cope with stress, and to support them in the management of experiences, even negative ones, as in this case.

Generally speaking, the vast majority of both mothers and fathers presented high levels of resilience and did not report stress symptoms at both T0 and T1. As observed for patients, it may also be that their parents present a kind of resistance to stress and/or challenge, which makes them prepared to cope with negative events. However, as previously stated for patients, future investigations involving a control group are needed in order to provide further support to this result.

Then, an inverse relationship emerged between the level of anxiety, depression, and stress experienced by parents, and their resilience. This result is consistent with the literature [43] and further highlights that the ability to thrive despite adversities and adapt to difficult situations can mitigate the negative effects of social distancing rules and general security measures on people’s mental health [22].

Over time, parents showed a significant decrease in the DASS-21 scale scores; more specifically, mothers presented a decrease in anxiety, and fathers in stress symptomatology. Therefore, as hypothesized, the relaxation of the measures to contain the infection led to an improvement in the psychological well-being of both mothers and fathers.

Despite the interesting findings that emerged from this work, some limitations need to be addressed. First, the small sample size, the prevalence of girls, and the inclusion of patients from a single neuropsychiatric unit in Italy did not allow for generalizing the observed results. Moreover, caution must be applied in interpreting results since the sample was mainly composed of patients with internalizing disorders. Another source for possible error which should be considered is the lack of a baseline measurement and so the retrospective collection of information about pre-emergency behaviors. An additional limitation of this study is the administration of self-report tools, which may be affected by several factors, such as social desirability, individual bias, or failure to understand questions. Finally, despite our preliminary analyses not highlighting significative differences for age and gender in the diagnostic categories, type of neuropsychiatric interventions during the lockdown, and psycho-behavioral profile defined through YSR/CBCL, further works with a larger sample size need to be carried out to clarify the role of such variables in influencing the adaptation and the psychological well-being of young patients during health emergencies.

## 5. Conclusions

In summary, this study explored the psychological well-being of a group of young Italian patients and their parents, in relation to the considerable and sudden changes caused by the COVID-19 pandemic and the related measures to contain the risk of infection.

The group of patients, mainly suffering from internalizing disorders, overall showed a good adaptation to the pandemic context. To confirm this, a greater psychological discomfort was detected in patients with behavioral problems, attributable to neurodevelopmental and conduct disorders, compared to patients with internalizing disorders at both T0 and T1.

Although our data are preliminary and require further investigations, they could have an impact on the reorganization of neuropsychiatric services required by the health emergency: the temporary suspension of usual and non-urgent clinical-care activities in person and the transition to telepsychiatry may be more suitable for certain psychopathologies, such as those characterized by internalizing symptoms (e.g., anxiety, depressive disorders, somatic complaints), while other conditions more impacting on a behavioral level should maintain specialist interventions in person, also guaranteeing more resources in terms of space and operators.

With reference to what has been explained for patients characterized by disorders in the emotional-affective sphere, with an inherent avoidance component, it will be important to monitor their psychological well-being at the time of return at school, since that period could be particularly challenging for this group.

As for the parental environment, both mothers and fathers showed a good adjustment to the difficult situation caused by the COVID-19 outbreak. Moreover, most parents presented high levels of resilience, which may have helped them to successfully face adverse circumstances. To support this, lower levels of stress, depression, and anxiety were assessed in parents with higher levels of resilience. Therefore, resilience was confirmed to be a significant protective factor against psychological maladjustment. This result could be particularly useful in implementing parenting support interventions, especially in periods characterized by uncertainty and burden, such as health emergencies. In fact, programs which foster resilience and promote the development of personal skills could represent a promising avenue to prevent the onset of parental stress and burn-out, thus also improving the psychological well-being of the whole family.

## Figures and Tables

**Table 1:** Mean (M) and Standard Deviation (SD) values in the main YSR and CBCL scales of patients at T0.

Scale	YSR (*N* = 41)	CBCL Mothers (*N* = 55)	CBCL Fathers (*N* = 55)
	M (SD)	M (SD)	M (SD)
Total Problems	57.9 (9.58)	63.0 (9.32)	60.1 (10.72)
Externalizing Problems	52.8 (8.21)	58.3 (9.83)	54.8 (10.21)
Internalizing Problems	60.4 (11.72)	66.9 (10.46)	62.3 (11.56)
PTSP	59.7 (7.19)	67.8 (11.03)	65.1 (10.31)

Notes: CBCL = Child Behavior Checklist; YSR = Youth Self-Report; PTSP = Post-Traumatic Stress Problems.

**Table 2:** Socio-demographic characteristics of parents at T0.

Socio-Demographic Characteristics		Mothers	Fathers
SES	Low (%)	27.3	2.3
	Medium-low (%)	13.6	20.5
	Medium (%)	13.6	31.8
	Medium-high (%)	34.1	36.4
	High (%)	11.4	9.1
Marital Status	Conjugated (%)	82.1	82.1
	Separated/divorced (%)	17.9	17.9
Working modalities	As usual (%)	37.3	41.2
	Working remotely (%)	23.5	31.4
	Interrupted (%)	39.2	27.5

Notes: SES = socio-economic status; low SES = score between 8 and 19, medium-low SES = score between 20 and 29, medium SES = score between 30 and 39, medium-high-SES = score between 40 and 54, high SES = score between 55 and 66.

**Table 3:** Mean (M) and Standard Deviation (SD) values in the DASS-21 scales of parents at T0.

DASS-21 Scale	Mothers	Fathers
	M (SD)	M (SD)
Depression	49.9 (9.95)	48.6 (9.83)
Anxiety	49.4 (13.5)	45.1 (11.5)
Stress	51.7 (10.2)	48.1 (9.73)

**Table 4:** Spearman’s ρ correlations between resilience values and the DASS-21 scale scores of mothers and fathers at T0.

	DEP Mother	ANX Mother	STRESS Mother	DEP Father	ANX Father	STRESS Father
Resilience 1	−0.51 **	−0.23	−0.43 **	−0.30 **	−0.45 **	−0.28
Resilience 2	−0.42 **	−0.36 *	−0.22	−0.40 *	−0.24	−0.40 *
Resilience 3	−0.43 **	−0.32 *	−0.60 **	−0.43 *	−0.31 *	−0.48 *

Notes: * *p* < 0.05; ** *p* < 0.003 (Bonferroni correction). DEP = DASS-21 Depression scale; ANX = DASS-21 Anxiety scale; STRESS = DASS-21 Stress scale; Resilience 1 = question 1 about commitment; Resilience 2 = question 2 about control; Resilience 3 = question 3 about challenge.

**Table 5:** Mean (M) and Standard Deviation (SD) values in the main YSR and CBCL scales of patients at T1.

Scale	YSR (*N* = 31)	CBCL Mothers (*N* = 39)	CBCL Fathers (*N* = 39)
	M (SD)	M (SD)	M (SD)
Total Problems	55.9 (9.06)	61.3 (9.27)	57.7 (10.3)
Externalizing Problems	51.2 (7.82)	56.1 (8.95)	53.9 (9.49)
Internalizing Problems	58.8 (11.09)	64.4 (10.06)	60.7 (11.4)
PTSP	59 (7.80)	64.3 (10.03)	62.4 (10.3)

Notes: CBCL = Child Behavior Checklist 6–18; YSR = Youth Self-Report 11–18; PTSP = Post-Traumatic Stress Problems.

**Table 6:** Mean (M) and Standard Deviation (SD) values in the DASS-21 scales of parents at T1.

DASS-21 Scale	Mothers	Fathers
	M (SD)	M (SD)
Depression	47.5 (7.01)	45.9 (7.39)
Anxiety	44.5 (7.35)	42.1 (3.99)
Stress	48.1 (7.94)	46.1 (7.67)

**Table 7:** Spearman’s ρ correlations between resilience values and the DASS-21 scale scores of mothers and fathers at T1.

	DEP Mother	ANX Mother	STRESS Mother	DEP Father	ANX Father	STRESS Father
Resilience 1	−0.31	−0.30	−0.30	−0.36 *	−0.22	−0.23
Resilience 2	−0.32	−0.28	−0.29	−0.25	−0.36 *	−0.39 *
Resilience 3	−0.08	−0.21	−0.36 *	−0.60 **	−0.34 *	−0.38 *

Notes: * *p* < 0.05; ** *p* < 0.003 (Bonferroni correction). DEP = DASS-21 Depression scale; ANX = DASS-21 Anxiety scale; STRESS = DASS-21 Stress scale; Resilience 1 = question 1 about commitment; Resilience 2 = question 2 about control; Resilience 3 = question 3 about challenge.

## Data Availability

Data presented in this study are available on reasonable request to the corresponding author. Data are not publicly available because they report private information about participants.
